# Protooncogene TCL1b functions as an Akt kinase co-activator that exhibits oncogenic potency *in vivo*

**DOI:** 10.1038/oncsis.2013.30

**Published:** 2013-09-16

**Authors:** M Hashimoto, F Suizu, W Tokuyama, H Noguchi, N Hirata, M Matsuda-Lennikov, T Edamura, M Masuzawa, N Gotoh, S Tanaka, M Noguchi

**Affiliations:** 1 Division of Cancer Biology, Institute for Genetic Medicine, Hokkaido University, Sapporo, Japan; 2 Department of Surgical Pathology, Toho University School of Medicine, Sakura, Japan; 3 Department of Pathology, Teine Keijinkai Hospital, Sapporo, Japan; 4 Department of Dermatology, Kitasato University School of Medicine, Sagamihara, Japan; 5 Division of Cancer Cell Biology, Cancer Research Institute, Kanazawa University, Kanazawa, Japan; 6 Department of Cancer Pathology, Hokkaido University Graduate School of Medicine, Sapporo, Japan

**Keywords:** protooncogene TCL1b, Akt, transgenic mice, angiosarcoma

## Abstract

Protooncogene T-cell leukemia 1 (TCL1), which is implicated in human T-cell prolymphocytic leukemia (T-PLL), interacts with Akt and enhances its kinase activity, functioning as an Akt kinase co-activator. Two major isoforms of *TCL1* Protooncogenes (*TCL1* and *TCL1b*) are present adjacent to each other on human chromosome 14q.32. In human T-PLL, both TCL1 and TCL1b are activated by chromosomal translocation. Moreover, TCL1b-transgenic mice have never been created. Therefore, it remains unclear whether TCL1b itself, independent of TCL1, exhibits oncogenicity. In co-immunoprecipitation assays, both ectopic and endogenous TCL1b interacted with Akt. In *in vitro* Akt kinase assays, TCL1b enhanced Akt kinase activity in dose- and time-dependent manners. Bioinformatics approaches utilizing multiregression analysis, cluster analysis, KEGG (Kyoto Encyclopedia of Genes and Genomes) pathway mapping, Venn diagrams and Gene Ontology (GO) demonstrated that TCL1b showed highly homologous gene-induction signatures similar to Myr-Akt or TCL1. TCL1b exhibited oncogenicity in *in vitro* colony-transformation assay. Further, two independent lines of β-actin promoter-driven TCL1b-transgenic mice developed angiosarcoma on the intestinal tract. Angiosarcoma is a rare form of cancer in humans with poor prognosis. Using immunohistochemistry, 11 out of 13 human angiosarcoma samples were positively stained with both anti-TCL1b and anti-phospho-Akt antibodies. Consistently, in various cancer tissues, 69 out of 146 samples were positively stained with anti-TCL1b, out of which 46 were positively stained with anti-phospho-Akt antibodies. Moreover, TCL1b structure-based inhibitor ‘TCL1b-*Akt-in*' inhibited Akt kinase activity in *in vitro* kinase assays and PDGF (platelet-derived growth factor)-induced Akt kinase activities—in turn, ‘TCL1b-*Akt-in*' inhibited cellular proliferation of sarcoma. The current study disclosed TCL1b bears oncogenicity and hence serves as a novel therapeutic target for human neoplastic diseases.

## Introduction

Serine threonine kinase Akt, also called Protein kinase B, was originally identified from the AKT8 acute transforming retrovirus that causes mouse thymoma.^1^ Genetic and functional alterations of the phosphatidylinositol-3 kinase-Akt signaling pathways underlie the pathogenesis of a wide variety of human diseases, such as neoplastic diseases, glucose intolerance, viral infection and autoimmune diseases.

The activation process of Akt is regulated by phosphorylation at two regulatory sites: threonine 308/309/305 and serine 473/474/472 (Akt1/2/3, respectively), with phosphorylation of both being required for maximum kinase activity. Both threonine 308 phosphorylation and membrane anchorage are required for serine 473 phosphorylation,^2^ which is required for complete activation of Akt.^3, 4^ Phosphoinositide-dependent protein kinase 1 has been identified as the primary kinase phosphorylating Akt on Thr308.^5^ The identity of the kinase(s)—putatively named phosphoinositide-dependent protein kinase 2—that are responsible for phosphorylation of the serine residue at 473/474/472 has recently been clarified as mTORC2 (mTOR, mLST8, SIN1 and Rictor complexes).^6, 7^

**Figure 1:**
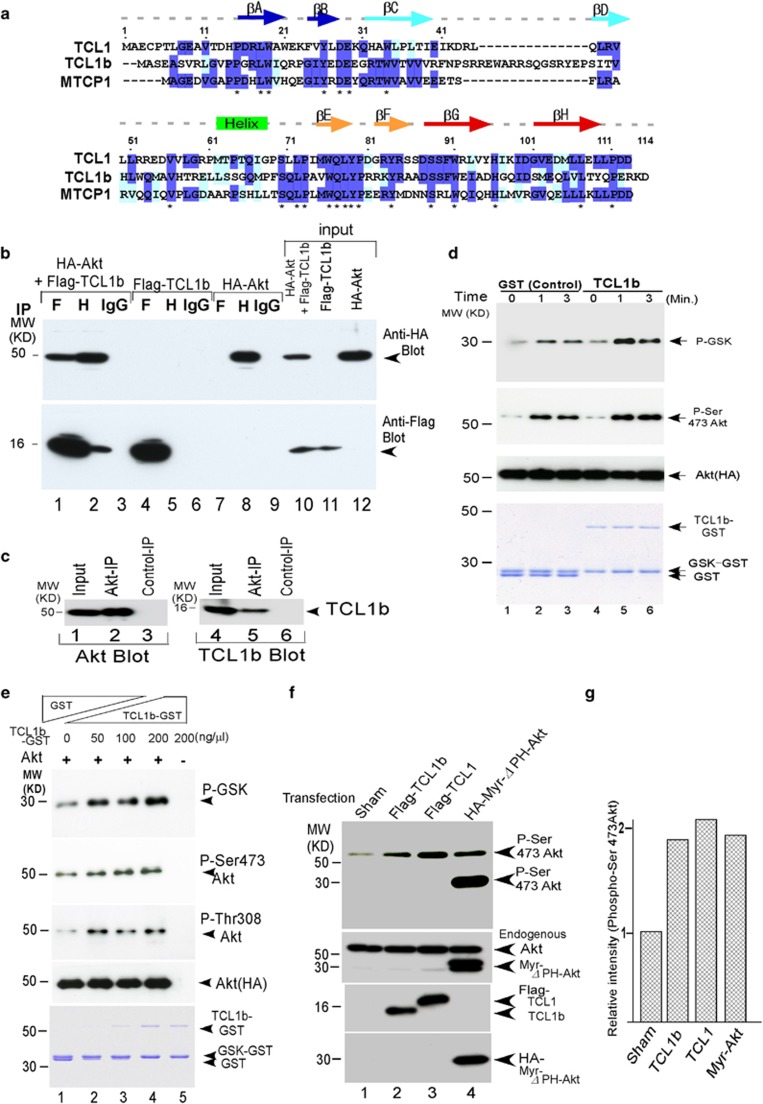
TCL1b interacted with Akt and enhanced Akt kinase activity. (**a**) Amino-acid sequence alignment of the human *TCL1* protooncogene family are shown using Clustal W (http://www.ch.embnet.org/software/ClustalW.html). (**b**) HA-tagged Akt2 and Flag-tagged TCL1b (as indicated) were transfected into 293T cells, and co-imunoprecipitation assays were conducted. HA-tagged Akt2 co-immunoprecipitated with Flag-tagged TCL1b in this assay. Single transfectants are shown as negative controls. There was no detectable background using control antibody. (H=anti-HA antibody; F=anti-Flag antibody). (**c**) In COS-7 cells, endogenous TCL1b interacted with Akt, albeit weakly, shown by using immunoprecipitation assays. (**d**) In *in vitro* kinase assays, presence of 9.6 μg of TCL1b-GST fusion proteins (0∼200 ng/μl) enhanced and promoted the kinetics of Akt-induced GSK-α phosphorylation (top panel; lanes 1–3;2866, 41852 and 36067 with control GST protein, lanes 4–6;3237, 55031 and 58123 with TCL1b). Analogously, Akt phosphorylation on Ser 473 was enhanced (second row; lanes 1-3; 18619, 26468 and 24667 with control GST protein, lanes 4–6; 14842, 55109 and 39965 with TCL1b protein). The amount of the TCL1b-GST protein or the control GST protein in the reactions is shown by coomassie brilliant blue staining (bottom row). Equal amounts of immobilized Akt were used as verified by using western blotting against HA (Third row). The levels of signal intensities were measured using NIH ImageJ. (**e**) The presence of 0–6.4 μg of TCL1b-GST fusion proteins in *in vitro* kinase assays enhanced Akt-induced GSK-α phosphorylation (top row), Akt phosphorylation of Ser473 (second row) and Thr308 (3rd row) in a dose-dependent manner. Equal amounts of immobilized Akt were used as verified by using western blotting against HA (fourth row). The amount of TCL1b-GST protein, GSK-GST and GST protein in the reactions were shown by using coomassie brilliant blue staining (bottom row). (**f**) Analogous to Myr-Akt (Myr-Akt (HA-Myr-*Δ*PH (4-129) human-Akt1 in pECE vector^54^), a constitutively active form of Akt, transfection of TCL1 or TCL1b into 293T cells enhanced Akt kinase phosphorylation at Ser 473. Equal amounts of Akt, each of the Flag-tagged proteins, and HA-tagged Myr-Akt were expressed as verified by using western blotting (lower panels, anti-Akt antibody, Flag antibody or HA antibody). Please note that as the PH domain is deleted in this Myr-Akt construct(HA-Myr-*Δ*PH (4-129) human-Akt1^54^), the size of the band on sodium dodecyl sulfate (SDS) gel exhibited in 30 kDa in size on SDS gels. (**g**) Quantitation of the signals of phospho-Ser 473 Akt in panel (**f**) is measured and displayed as bar graph using NIH ImageJ.

The protooncogene *TCL1* (T-cell leukemia 1) family protein was first identified in the translocation of T-cell prolymphocytic leukemia (T-PLL), a rare form of human adulthood leukemia.^8, 9^ The physiological expression of TCL1 is tightly limited to early developmental cells as well as various developmental stages of immune cells. Expression of TCL1 is also observed in germ cell neoplasia and seminoma.^10^ However, the biological function of protooncogene TCL1 was poorly understood until we had demonstrated that protooncogene TCL1 physically interacts with Akt and enhances Akt kinase activity through transphosphorylation mechanisms, thus functioning as an Akt kinase co-activator.^11, 12, 13, 14^ Three TCL1 family proteins are known to be present in human genome, namely TCL1, TCL1b and MTCP1—which consist of 114, 128 and 106 amino acids, respectively—with predicted molecular weight of 14 (TCL1), 15 (TCL1b) and 13 kDa (MTCP1). These TCL1 isoforms share a relatively high sequence homology (Figure 1a). X-ray crystallographic analysis of TCL1 suggested that TCL1 family proteins featured a unique symmetrical β-barrel structure.^15, 16^

In human T-PLL, both *TCL1* and *TCL1b* genes are activated by juxtaposition onto the T-cell receptor α or β loci, secondary to chromosomal translocations t(14:14) (q11: q32), t(7:14)(q35: q32) or inversion (14)(q11: q32).^17, 18^ TCL1- or MTCP1-deregulated mice exhibited neoplastic diseases of immune systems;^19, 20, 21^ however, TCL1b-deregulated mice have never been created. Therefore, it remains unclear whether TCL1b itself, independent of TCL1, bears oncogenicity, *in vivo*, underlying human neoplastic diseases.

In this study, we investigated whether and how TCL1b functions as an Akt kinase co-activator and exhibits oncogenic potency, both *in vitro* and *in vivo*, underlying various human neoplastic diseases. With the help of biochemical approaches, we showed that TCL1b physically interacts with Akt and enhances Akt kinase activity. Applying bioinformatics approaches of cluster analysis, Venn diagram, KEGG (Kyoto Encyclopedia of Genes and Genomes) pathway mapping and Gene ontology (GO) using the Agilent DNA microarray analysis demonstrated that highly homologous gene-induction signatures were obtained among TCL1b, Myr-Akt and TCL1. TCL1b showed potent oncogenicity both *in vitro* and in deregulated mice, which resulted in angiosarcoma of the intestinal tract. Consistently, human angiosarcoma samples and human cancer tissue array were positively stained with anti-TCL1b and anti-phospho-Akt antibodies. Moreover, TCL1b structure-based inhibitor, namely ‘TCL1b-*Akt-in'*, efficiently inhibited platelet-derived growth factor (PDGF)-stimulated Akt kinase activity, which, in turn, inhibited cellular proliferation of angiosarcoma and reticulum cell sarcoma. Collectively, observation disclosed that TCL1b indeed functions as an Akt kinase co-activator, underlies neoplastic diseases *in vivo* and, therefore, possibly serves as a novel therapeutic target of human neoplastic diseases.

## Results

### Protooncogene TCL1b interacted with Akt and enhanced Akt kinase activity

Relatively little is characterized about the pathophysiological roles of TCL1b, another major member of the TCL1 family protooncogene, in human neoplastic diseases.^17, 18^ In order to clarify the pathological functions of TCL1b underlying human neoplastic diseases, we first examined whether TCL1b can physically interact with Akt in mammalian cells. Co-immunoprecipitation assays were conducted using 293T cells with both Flag-TCL1b and HA-Akt transfected and demonstrated physical interaction of TCL1b with Akt (Figure 1b). Consistent with this finding, we showed that endogenous TCL1b interacted with Akt in COS-7 cells, which express endogenous TCL1b, using co-immunoprecipitation assays (Figure 1c).

The observation prompted us to study whether TCL1b-Akt interaction can be functional to enhance Akt kinase activity. We undertook *in vitro* Akt kinase assays, which demonstrated that in the presence of TCL1b, but not in the absence of TCL1b, Akt kinase activity was enhanced in a time-dependent (Figure 1d) and a dose-dependent manner (Figure 1e).

TCL1b, TCL1 or Myr-Akt, a constitutively active form of Akt as a positive control, was transfected into 293 cells. Phospho-specific immunoblot demonstrated that the levels of Akt phosphorylation at Ser473 were potently augmented in TCL1b and TCL1 at similar extent as Myr-Akt-transfected cells (Figures 1f and g). The results together supported the notion that analogous to TCL1, protooncogene TCL1b indeed functions as an Akt kinase co-activator that interacts with Akt, and hence enhances Akt kinase activity.

### Bioinformatic approaches revealed that TCL1b exhibited similar gene-induction signature as TCL1 or Myr-Akt, a constitutive active form of Akt

**Figure 2:**
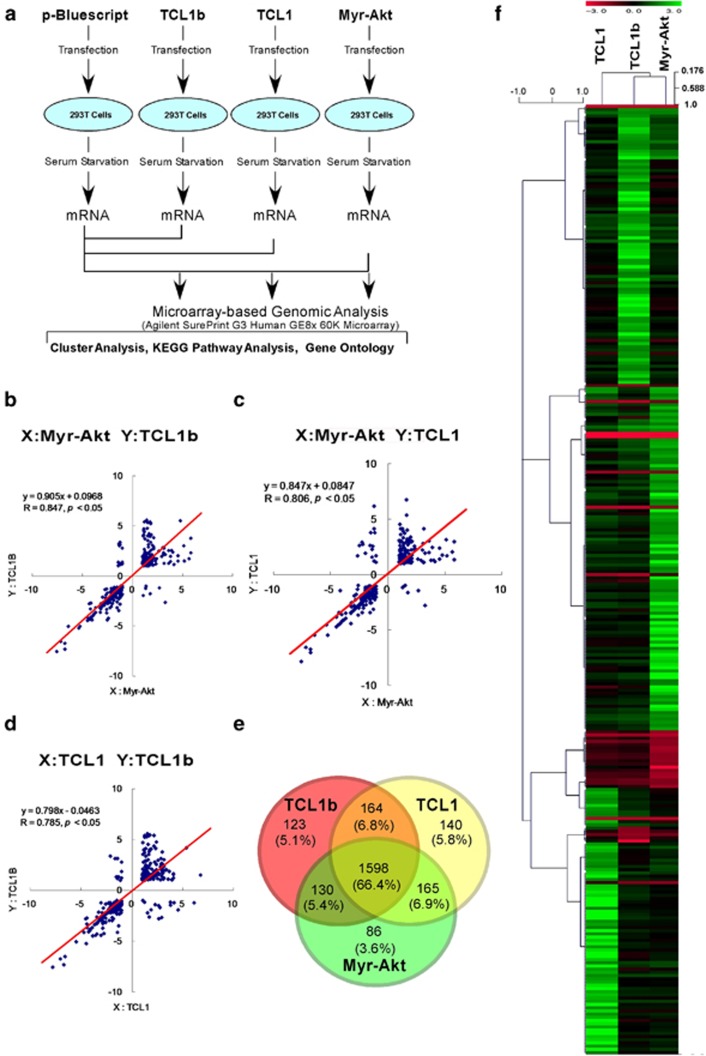
The Agilent Expression Array analysis indicated that TCL1b showed highly homologous gene-induction signatures to TCL1 or Myr-Akt—a constitutively active form of Akt. (**a**) Human TCL1b, TCL1 and Myr-Akt (HA-Myr-ΔPH (4-129) human-Akt1 in pECE vector^54^), a constitutively active form of Akt, with pBluescript (empty vector) as a baseline control were transfected into 293T cells. The cells were then serum-starved for 16 h and mRNAs were isolated from the cells for microarray-based genomic analysis using Agilent SurePrint G3 Human GE8 × 60K Microarray. Transcripts of either over two-fold increase or two-fold decrease compared with the control (pBluescript)-transfected cells as a baseline control were chosen for further analysis (**b**–**f**). (**b**–**d**) *R* value of the correlation coefficient of the regression analysis between TCL1b and Myr-Akt was 0.847 (*P*<0.05), between TCL1 and Myr-Akt was 0.806 (*P*<0.05) and between TCL1b and TCL1 was 0.785 (*P*<0.05). (**e**) The Venn diagram shows that 66.4% of the gene transcripts were simultaneously upregulated by TCL1, TCL1b and Myr-Akt, the constitutively active form of Akt. All gene transcripts analyzed by the Venn diagram includes either over two-fold increase or decrease by TCL1, TCL1b or Myr-Akt compared with the control (pBluescript)-transfected cells as a baseline control using GeneSpring 12 software (Agilent Technologies). (**f**) Heat map showing the hierarchical clustering analysis of TCL1b, TCL1 and Myr-Akt to classify the data into groups of genes with similar patterns that are characteristic to the group using MeV version 4.71. The distances of the clustering were calculated by using Pearson Correlation with Complete linkage.

Microarray-based technology using DNA microarrays has become an indispensable tool to monitor genome-wide expression levels of genes in various organisms. Thus, bioinformatic analysis can be a powerful tool for genome annotation—a general aspect of genomics. As Akt and TCL1 family proteins are known to regulate transcription factors such as FOXO family and NFκB family proteins,^22, 23, 24^ we utilized the microarray-based bioinformatic analysis of GO, KEGG mapping, Venn diagrams and cluster analysis to compare the global gene-induction signature of TCL1b, TCL1 and Myr-Akt. DNA microarray-based bioinformatics analysis was used to compare the gene-induction signature of TCL1b, TCL1 or Myr-Akt with empty vector (pBluescript)-transfected cells as a baseline control (Figure 2a). Gene transcripts induced by either a greater than two-fold increase or two-fold decrease compared with the pBluescript-transfected cells in microarrays were selected for further bioinformatics analysis. Raw data from the DNA microarray used for the bioinformatics analysis are presented in the Supplementary Data (Supplementary Data Table 1).

Microarray-based regression analysis provides much valuable information for modeling and analyzing several variables when the focus is on the relationship between a dependent variable and one or more independent variables. *R* values for correlation coefficient between TCL1b with Myr-Akt, TCL1 with Myr-Akt or TCL1b with TCL1 were 0.847, 0.806 or 0.785, respectively (Figures 2b–d). Furthermore, multiple regression analysis of the gene transcripts induced (or suppressed) by TCL1b, TCL1 or Myr-Akt revealed significant correlations between each other with *R* value of 0.865 (*P*<0.05) for the predicted equation of [Y (TCL1b)=0.295 × (TCL1)+0.655Z (Myr-Akt)+0.072] (Supplementary Data Figure S1 and Movie). The results together indicated that TCL1b, Myr-Akt and TCL1 induced significantly homologous groups of gene transcripts.

Venn diagrams are widely used to illustrate relationships in probability, logic and statistics of the gene-induction signature of different categories in various bioinformatic analyses. We analyzed gene profiles induced by TCL1b, TCL1 or Myr-Akt using Venn diagrams, which revealed that 66.4% of the overlapping gene transcripts were simultaneously upregulated (more than two-fold increase) by TCL1b, TCL1 and Myr-Akt (Figure 2e). Similar to this observation, 37.0% of the overlapping gene transcripts were simultaneously downregulated (more than two-fold decrease) by TCL1b, TCL1 and Myr-Akt. These observations supported the notion that TCL1b indeed induced highly homologous sets of gene transcripts as TCL1, or Myr-Akt, a constitutively active form of Akt.

DNA microarray-based cluster analysis provides powerful tools for genome annotation to build groups of genes with related expression patterns in the transcriptomics analysis.^25^ We utilized cluster analysis to compare the transcripts of TCL1, TCL1b and Myr-Akt with pBluescript-transfected cells as a baseline control. A heat map of the cluster analysis indicated that gene-induction signature from the cells transfected with TCL1, TCL1b or Myr-Akt resembled each other (Figure 2f), supporting the notion that TCL1b indeed enhanced Akt kinase activity to mediate similar down-stream intracellular signals as Myr-Akt or TCL1.

KEGG (http://www.kegg.jp/ja/) pathway mapping of the bioinformatics analysis is a collection of pathway maps including metabolism, genetic information processing, environmental information processing, cellular processes, organismal systems and various human diseases.^26, 27^ Simultaneously altered transcripts present in the MAPK cascades of the KEGG pathway by TCL1b, TCL1 and Myr-Akt included PDGF, PDGF receptor, fibroblast growth factor, calcium channel voltage-dependent gamma subunit, cytosolic phospholipase A2 and MKP (dual specificity phosphatase) (Supplementary Data Figures S2A–C).

The pathway of KEGG mapping in cancer also exhibited highly similar gene-induction signatures with TCL1b, TCL1 or Myr-Akt. Out of 327 total genes listed in the cancer pathway of KEGG, 16 genes were altered by TCL1b, 20 genes by Myr-Akt and 15 genes by TCL1. The genes simultaneously altered with TCL1b, TCL1 and Myr-Akt included Bcl-2, Cyclin D1, fibroblast growth factors, PDGFR, MCSFR (macrophage colony- stimulating factor receptor), AM1ETC (runt-related transcription factor 1), AMLEVI I (MDS1 and EVII complex locus runt-related transcription factor 1) or AML1 (runt-related transcription factor 1) in the cancer pathway of KEGG pathway mapping. This observation further supported that TCL1b indeed activated similar sets of gene transcripts as Myr-Akt or TCL1 to regulate cellular responses in the cancer pathway (Supplementary Figures S3A–C).

GO (http://www.geneontology.org) provides an unbiased biological gene-enrichment analysis based on biological properties (GO terms) assigned per individual gene product.^28^ Overall profiles categorized by GO slim closely resembled each other among TCL1b-, TCL1- and Myr-Akt-transfected cell-derived transcripts in all three categories (molecular function, cellular component or biological process) (Supplementary Data Figures S4A–C).Collectively, TCL1b altered similar functional groups of gene transcripts as TCL1, or Myr-Akt, the constitutively active form of Akt.

### TCL1b promoted oncogenic potential in soft agar colony-transformation assays

Similar gene-induction profiles with TCL1b, TCL1 or Myr-Akt in the KEGG pathways, GO slims and cluster analysis prompted us to further examine whether TCL1b bears abilities for transforming viable cells analogous to Myr-Akt, a constitutively active form of Akt,^29, 30^ or TCL1.

**Figure 3:**
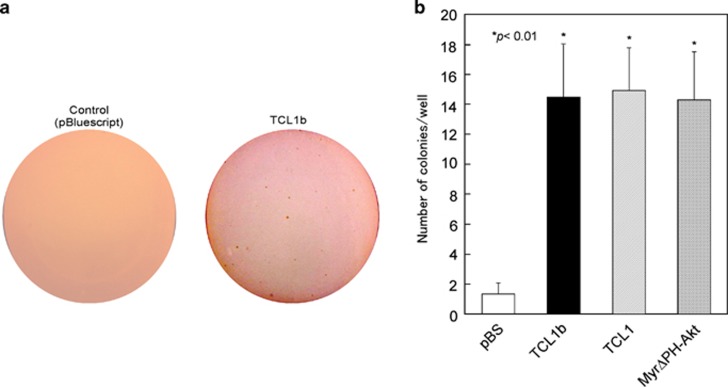
TCL1b exhibited oncogenicity in *in vitro* soft agar colony-transformation assays. (**a**) NIH3T3 cells were transiently transfected with expression vectors for TCL1b, Myr-Akt, TCL1 or pBluescript and used for the soft-agar colony-transformation assay after 21 days for microscopic analysis of colony formation. (**b**) The numbers of the transformed colonies with manual counting of proliferated NIH3T3 cell obtained from TCL1b-, Myr-Akt- (HA-Myr-ΔPH (4-129) human-Akt1 in pECE vector^54^), TCL1- or pBluescript-transfected NIH3T3 cells were 14.3, 14.7, 14.0 and 1.3, respectively.

For this purpose, a soft agar colony-transformation assay was conducted using NIH3T3 cells transfected with TCL1b, TCL1 or Myr-Akt with pBluescript-transfected cells as a negative control. The numbers of the transformed colonies obtained in this assay from TCL1b-, Myr-Akt-, TCL1- or pBluescript-transfected cells were 14.3, 14.7, 14.0 and 1.3, respectively (Figures 3a and b). This observation established that TCL1b exhibits oncogenicity *in vitro*.

### β-actin-driven TCL1b-transgenic mice resulted in angiosarcoma

**Figure 4:**
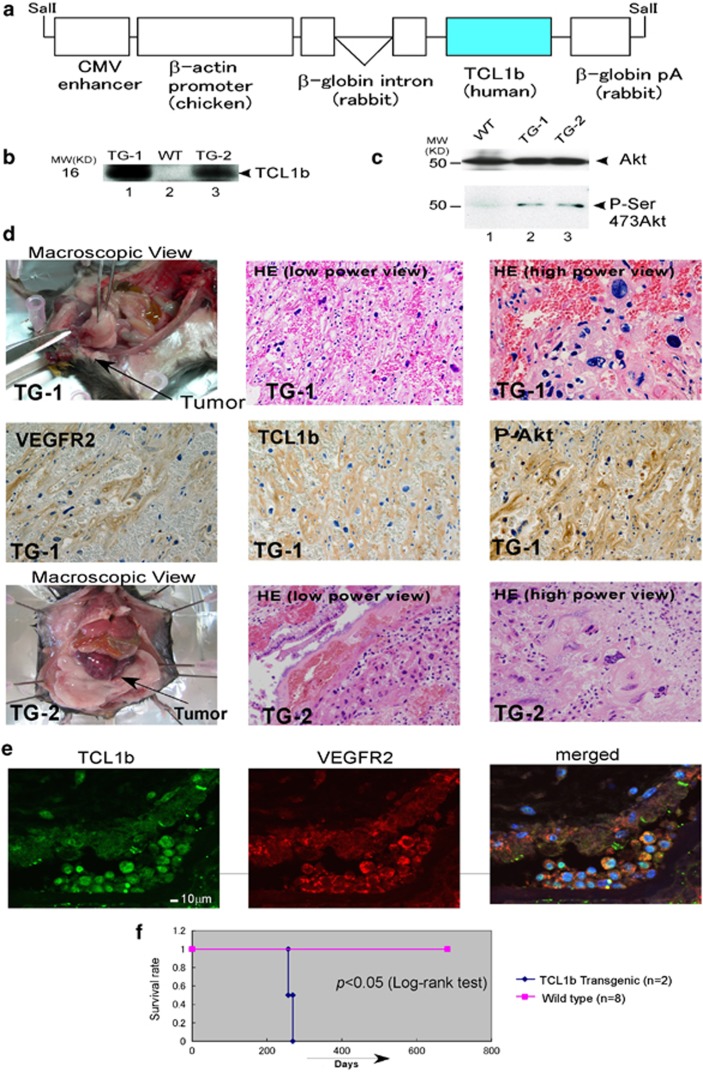
TCL1b-transgenic mice developed angiosarcoma. (**a**) CMV-β-actin-driven TCL1b-transgenic constructs (pUC-CAGGS-TCL1b^31^) were shown schematically. (**b**) The expressions of TCL1b of the two independent lines of transgenic mice were also confirmed by using immunoblot. (**c**) The levels of phospho-Akt of the muscle tissue from the two lines of transgenic mice are shown by anti-phospho-Akt (Ser473) immunoblot (lower panel) with anti-Akt immunoblot as an internal control (upper panel). (**d**) After 8 months of DOB, two independent lines of β-actin promoter-driven TCL1b-transgenic mice developed angiosarcoma in the intestinal submucosal tissues, which formed a huge, well-demarcated submucosal mass. Macroscopic view and HE staining (low-power view and high-power view) of the two independent lines of transgenic mice are shown (TG-1 and TG-2, top and bottom row, respectively). Microscopically, the tumor cells form irregular anastomosing vascular channels, have large nuclei and prominent nucleoli, as observed using HE staining. Immunohistochemically, the tumor cells were positively stained with anti-VEGFR2, TCL1b and phospho-Ser-473Akt antibodies (P-Akt) (middle panels). (**e**) TCL1b is expressed with VEGFR2, as determined by the co-staining of anti-VEGFR and anti-TCL1b antibodies and examined using confocal microscopy. (**f**) Kaplan–Meier curves of the transgenic lines using wild type as a control are shown. Statistically significant difference of the survival rate is observed between wild-type and TCL1b-transgenic lines (*P*<0.05, log-rank test).

Previously, immune lineage-specific TCL1- or MTCP1-transgenic mice have been created, which exhibited lineage-specific neoplastic changes.^19, 20, 21^ However, TCL1b-transgenic mice have never been reported. As the physiological expression of TCL1b is not restricted to immune cell-specific lineages,^17, 18^ we have chosen β-actin promoter (pUC-CAGGS-TCL1b^31^) for ubiquitous overexpression of TCL1b on C57BL/6 background for creating transgenic mice (Figure 4a).

As predicted, TCL1b-deregulated mouse tissues from the muscle showed elevated levels of phospho-Akt using immunoblot and TCL1b (Figures 4b and c). After 8 months of DOB, two independent lines of transgenic founder mice developed angiosarcoma in intestinal submucosal tissues. Macroscopically, in both transgenic founder lines, the neoplasm formed a huge, well-demarcated submucosal mass projecting intraluminally, presumably causing fatal obstruction of the intestine (Figure 4d). Microscopically by using HE staining, irregular anastomosing vascular channels were lined by highly atypical spindled endothelial cells with mitotic activity. Immunohistochemistry revealed that tumor tissues were stained positive by anti-TCL1b antibody (middle panels and please also see Supplementary Data Figure S5) and anti-phospho-Akt antibody (right side panels). In sarcoma tissues positively stained by both anti-VEGFR2 (vascular endothelial growth factor receptor 2) and TCL1b by confocal microscopy (Figure 4e). Positively stained tumor tissues by anti-VEGFR2 antibody confirmed that the tumor was indeed arising from vascular endothelial origin. Moreover, survival curve of the Kaplan-Meyer curve of the transgenic lines showed statistically significant early death compared to the wild type mice (Figure 4f).

### Human angiosarcoma samples were positively stained with both anti-TCL1b and anti-phospho-Akt antibodies

**Figure 5:**
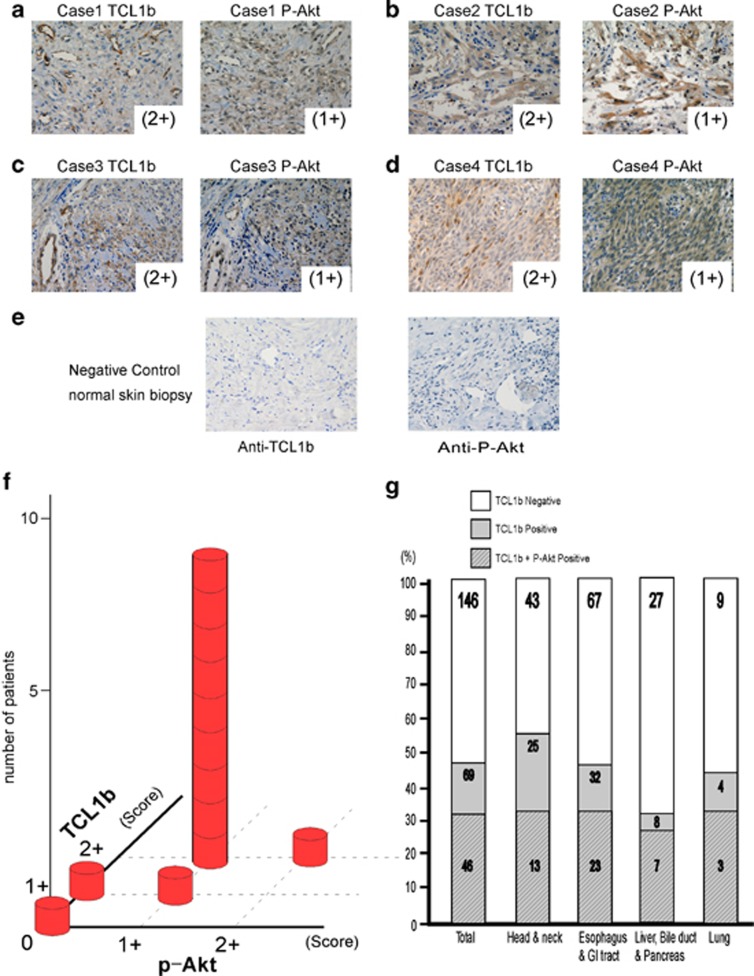
TCL1b and p-Akt expression of human angiosarcoma and cancer tissues. (**a**–**d**) Using immunohistochemistry, human angiosarcoma tissues were positively stained with the anti-TCL1b antibody and anti-phospho-Akt. Shown here are four representative cases of double-positive samples. (**e**) Negative control of anti-TCL1b and anti-P-Akt were shown by immunostaining of normal skin biopsy. (**f**) Correlation of anti-TCL1b and anti-phospho-Akt of human angiosarcoma tissue samples is shown as a bar graph. Eleven out of 13 cases were positive in both anti-TCL1b and anti-P-Akt immunostaining. (**g**) Cancer tissue arrays of 146 human cancer tissues (SuperBioChips laboratories) were examined by using immunohistochemistry with anti-TCL1b-specific and anti-phospho-Akt antibodies. Among the 69 cases of TCL1b-positive cancer tissues, 46 cases (67%) were stained positive with anti-phospho-Akt antibody.

Human angiosarcoma is a rare, rapidly growing sarcoma, which is highly invasive and arises from endothelial cells.^32^ Having demonstrated that mouse-deregulated TCL1b expression resulted in angiosarcoma, we next examined human angiosarcoma tissues by immunostaining with anti-TCL1b or phospho-Akt antibodies. Indeed, by using immunohistochemistry, 11 out of 13 cases of human angiosarcoma were stained positive with both anti-TCL1b and phospho-Akt antibodies (Figures 5a–f). The observation supported the notion that TCL1b possibly has an active role in the pathological condition of angiosarcoma in humans.

### Human cancer tissue array stained positive with anti-TCL1b and anti-phospho-Akt antibodies

In contrast to TCL1, physiological expression of TCL1b is not limited to immune tissues.^17, 18^ This observation prompted us to hypothesize that unlike TCL1, TCL1b may have a role in oncogenesis of non-lymphoid organs. To further elucidate the pathological role of TCL1b in neoplastic diseases of non-lymphoid origin, human cancer tissue panels (SuperBioChips laboratories, Seoul, South Korea) were examined using the TCL1b-specific antibody (Supplementary Data Figure S5). In this analysis, 69 cases out of 146 (47.3%) cancer tissue samples showed positive results with the TCL1b antibody. TCL1b was positively stained in 25 of 43 (58.1%) head and neck cancer types, 32 of 67 (47.8%) esophagus and gastric cancer types, 8 of 27 (29.7%) liver, bile duct and pancreas, and finally in 4 of 9 (44.4%) lung cancer types. Notably, among the 69 cases of TCL1b-positive cancer tissues, 46 cases (67%) were stained positive simultaneously with the anti-phospho-Akt antibody (Figure 5g). The observations together supported the notion that TCL1b, which activates Akt, possibly underlies various human cancer types of non-lymphoid lineages.

### TCL1b structure-based inhibitor ‘TCL1b-Akt-in' suppressed Akt kinase activity and cellular proliferation of sarcoma or cancer cells

**Figure 6:**
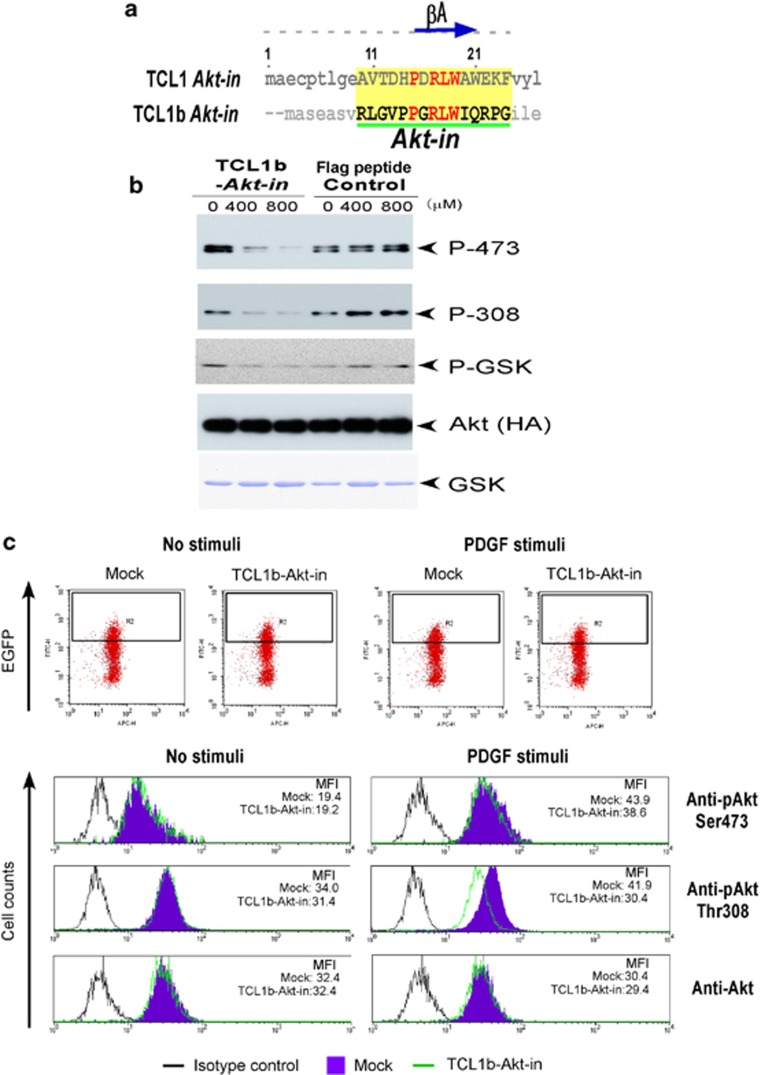
TCL1b-based inhibitor ‘TCL1b-Akt-in' compromised Akt kinase activity. (**a**) Amino-acid sequence alignment of TCL1-based Akt inhibitor ‘TCL1-*Akt-in'* and TCL1b-based Akt inhibitor ‘TCL1b-*Akt-in'* are shown. Conserved amino acids between TCL1 and TCL1b are highlighted in red. (**b**) In *in vitro* Akt kinase assays using GSK-α as a substrate, ‘TCL1b-*Akt-in*' drastically inhibited Akt kinase activity in a dose-dependent manner. (**c**) Retroviral transduction of NIH3T3 cells with TCL1b-*Akt-in* was compared with pBMN-GFP plasmids (Mock) using the Phoenix Retroviral Expression System (Orbigen). The levels of the Phospho-Akt Ser473 (4060, Cell Signaling Technology), Phospho-Akt Thr308 (2965, Cell Signaling Technology) or Total Akt (9272, Cell Signaling Technology) of GFP-positive cells were assessed by using flow cytometer gated on the GFP-positive cells shown in the right upper panels (FACS CantoII, BD Biosciences).

**Figure 7:**
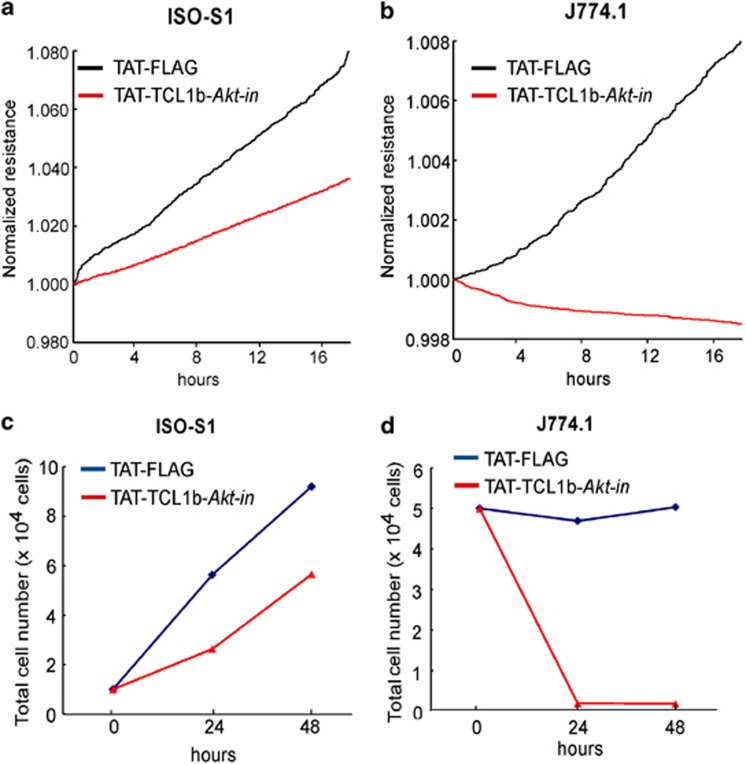
TCL1b-based inhibitor ‘TCL1b-Akt-in' inhibited cell growth in human and mouse sarcoma cells. (**a** and **b)** Growth potentials of ISO-S1 (mouse angiosarcoma) and J774.1 (mouse reticulum cell sarcoma) were assessed by using Electric Cell-Substrate Impedance Sensing (ECIS Zθ Applied Biophysics). J774.1 and ISO-HAS-B-cell lines were seeded in 8WCP cell-proliferation plates with 50 μM of TAT-TCL1b-*Akt-in* peptide or TAT-FLAG as a control. (**c** and **d**) Growth potentials of ISO-S1 (mouse angiosarcoma) and J774.1 (mouse reticulum cell sarcoma) were assessed by trypan blue staining and by counting the cell numbers under microscopy. For this purpose, ISO-S1 (1 × 10^4^ cells) and J774.1 cells (5 × 10^4^ cells) were seeded in 24-well plates with 50 μM of TAT-FLAG (control) or TAT-TCL1b-Akt-in peptide. Cells were counted using trypan blue dye exclusion method at 24 and 48 h after peptide treatment.

On the basis of the structural functional analysis of the TCL1-Akt protein complex, we have previously identified and characterized a TCL1 structure-based peptide, named ‘*Akt-in*' (Akt inhibitor, NH_2_-AVTDHPDRLWAWEKF-COOH encompassing the βA strand of TCL1), interacted with Akt and specifically inhibited its kinase activity and proliferation of cancer cells.^11, 33^ The amino-acid sequences of the protooncogene TCL1 and TCL1b share a relatively high sequence homology with a possible common three-dimensional structure. Therefore, we reasoned to create a TCL1b structure-based peptide inhibitor of the putative Akt-binding domain, namely ‘TCL1b-*Akt-in*' (Figure 6a), to examine the Akt inhibitory effect of the peptide. Indeed, ‘TCL1b-*Akt-in*' effectively inhibited Akt kinase activity both *in vitro* (Figure 6b) and in the cell (Figure 6c). Moreover, as predicted by the inhibition of Akt kinase activity, ‘TCL1b-*Akt-in*' inhibited cellular proliferation of angiosarcoma or reticulosarcoma (Figures 7a–d). The observation together strongly supported that the protooncogene TCL1b is a novel molecular target with therapeutic potential for human cancer types in clinical settings.

## Discussion

We have previously demonstrated that protooncogene TCL1, implicated in human T-PLL chronic adulthood leukemia, is an Akt kinase co-activator.^11, 12, 13, 14^ TCL1 physically binds to Akt and activates Akt via a transphosphorylation reaction.^11, 34, 35^

The *TCL1b* gene is located adjacent to the *TCL1* oncogene in human chromosome 14q.32. The physiological expression of TCL1 family members is mainly restricted to lymphoid tissues; however, *TCL1b* mRNA is not restricted to lymphoid lineages, including early stages of embryos and placenta.^17, 18^ TCL1 and TCL1b are highly expressed at early developmental stages in several fetal tissues, including the thymus, kidney, lung (TCL1) and spleen (TCL1b).^9, 18, 20, 36, 37, 38^

Although pathophysiological roles of TCL1 are primarily investigated in immunological malignancies including human T-PLL,^39, 40^ AIDS-related lymphoma, and/or B-cell lymphoma,^14, 41^ knowledge is limited about pathophysiological roles of the TCL1 family proteins in non-lymphoid tissues or neoplastic diseases. As TCL1 family protooncogenes were shown to enhance Akt kinase activity, it is logical to speculate that analogous to the pathophysiological roles of Akt,^42^ TCL1 family protooncogenes could also be underlying the pathogenesis of human cancer types of the non-lymphoid origin. As predicted by the highly homologous amino-acid sequences among the TCL1 family proteins (see Figure 1a), TCL1b also forms dimers in co-immunoprecipitation assays, suggesting that TCL1b enhances Akt kinase activity via dimerization-mediated transphosphorylation analogous to the mechanisms of TCL1 to enhance Akt kinase activity.^11, 34, 35^

Deregulation of TCL1 and MTCP1 in mouse immune cells resulted in lineage-specific leukemia of lymphoid cells.^19, 20, 39, 40^ However, up-to-date TCL1b-transgenic mice have never been reported even in an immune cell lineage-specific manner. Striking observations were revealed that at 8 months of age two independent lines of β-actin promoter-driven TCL1b-transgenic mice developed angiosarcoma arising in intestinal tissues that share some characteristic morphological features of human angiosarcoma.

Human angiosarcoma is a rare soft-tissue sarcoma of the endothelial cell origin with poor prognosis. In humans, angiosarcoma can occur essentially anywhere in the body. However, it is most commonly presented as a cutaneous disease, which involves the head and neck and particularly the scalp in elderly white men. No comprehensive studies of molecular changes in angiosarcoma have been conducted; however, specific chromosomal abnormality of angiosarcoma seems not to be involved including the TCL1b locus of human chromosome 14q.32.^43^ Formation of soft-tissue sarcoma was reported in 36% of Eμ-TCL1-transgenic mice. In contrast to our study, Zanesi *et al.*^44^ did not find TCL1 expressed in the tumor tissues; therefore, they speculate that soft-tissue sarcoma was occurring because of secondary malignancies in immunocompromised status. Notably, Akt activation is known to be associated with angiosarcoma of human, mice and chickens.^29, 30, 45, 46^ Furthermore, microvascular patterning is known to be controlled by fine-tuning the Akt-mediated signal transduction.^47^ Consistently, sustained signaling in response to the overexpression of Myr-Akt leads to embryonic lethality, edema and vascular malformations. The vascular phenotype is consistent with a failure in remodeling such that normal patterning and vessel hierarchy were disturbed.^48^ Endothelial cells in growing tumors express activated Akt, which when modeled by transgenic endothelial expression of Myr-Akt were sufficient to recapitulate the abnormal structural and functional features of tumor blood vessels in nontumor tissues. Furthermore, sustained endothelial Akt activation is known to cause increased blood vessel size and generalized edema from chronic vascular permeability.^49^ Consistent with the reports that high levels of phosphorylated Akt is observed in angiosarcoma,^46, 50, 51^ angiosarcoma derived from the TCL1b-deregulated mice showed to be positive with phospho-Akt using immunohistochemistry. Furthermore, we showed that 11 out of the 13 cases of human angiosarcoma stained positive with both phospho-Akt and TCL1b using immunohistochemistry. The observation supported that TCL1b-mediated activation of Akt may underlie the malignant transformation of generation of angiosarcoma. Notably, immunohistochemistry of human cancer array showed 69 out of 146 human cancer derived from various tissue origins appeared positively stained with the anti-TCL1b antibody. It is of note that in human cancer tissue arrays, positive staining of TCL1b appeared to show no obvious correlations with clinical stages of human cancer types.

Microarray-based bioinformatics analysis allowed us to monitor the expression level of genes at a genome scale, which enables us to cluster genes with similar expression profiles together to make meaningful biological inferences about the global set of gene transcripts. As Akt and TCL1 family proteins are known to regulate transcription factors such as FOXO family and NFκB family proteins,^22, 23, 24^ we utilized the microarray-based bioinformatics analysis of GO, KEGG mapping, Venn diagrams and cluster analysis to compare the global gene-induction signature of TCL1b, TCL1 and Myr-Akt. Indeed, highly homologous gene-induction signatures observed using bioinformatics analysis, in particular between TCL1b and Myr-Akt, supported the notion that TCL1b not only functions as an Akt kinase co-activator but also has an active role in the pathogenesis of various human neoplastic diseases—in particular angiosarcoma.

In an attempt to develop Akt-specific inhibitors based on the Akt–TCL1 protein complexes, we have identified a peptide inhibitor, which spans the βA sheet of TCL1, consisting half sheet of the Akt-binding domain for TCL1, named ‘TCL1-*Akt-in'* (Akt inhibitor, NH2-AVTDHPDRLWAWEKF-COOH), which retained the interaction with Akt but lacked the ability for oligomerization. As was revealed using nuclear magnetic resonance chemical mapping, the ‘TCL1-*Akt-in'* peptide inhibitor bound the Akt-PH in a surface similar to that recognized by TCL1 proteins. Mechanically, interaction of ‘TCL1-*Akt-in'* with the Akt-PH domain prevented phosphoinositide binding to the Akt-PH domain and, hence, inhibited membrane translocation and activation of Akt. Functionally, ‘TCL1-*Akt-in'* inhibited not only cellular proliferation and anti-apoptosis *in vitro* but also *in vivo* tumor growth.^33^ Further, cellular proliferation of human cancer cell lines was effectively inhibited *in vitro* by ‘TCL1-*Akt-in'*.^11, 33, 52^

Having demonstrated that the deregulation of TCL1b resulted in angiosarcoma and positive immunohistochemistry of TCL1b in human angiosarcoma and various human cancer types, we attempted to design the inhibitory peptides based on the amino-acid sequences and the three-dimensional predicted structure of TCL1b. Indeed, TCL1b structure-based *Akt-in* ‘TCL1b-*Akt-in'*, RLGVPPGRLWIQRPG, corresponding amino-acid sequences of TCL1-based *Akt-in* are underlined) was designed, which inhibited not only Akt kinase activity in a dose-dependent manner in *in vitro* kinase assays, but also PDGF-stimulated Akt kinase activity. Notably, ‘TCL1b-*Akt-in'* effectively inhibited the cellular proliferation of angiosarcoma.

Given the highly positive immunostaining of TCL1b in human angiosarcoma and various human cancer tissues, it is plausible that TCL1b can serve as a putative molecular target for neoplastic diseases in general, in particular targeting angiosarcoma in humans.

As TCL1b expresses ubiquitously as was suggested by the housekeeping type of 5′-promoter sequences, we have created β-actin-driven TCL1b-transgenic mice, which resulted in angiosarcoma in the intestinal submucosal tissues. Angiosarcoma is a rare form of human sarcoma with poor prognosis. Phosphatidylinositol-3 kinase-Akt pathways are shown to be activated in angiosarcoma; however, no comprehensive studies have been conducted for elucidating the molecular etiology of the diseases.^43^ No therapeutic intervention has been developed for the treatment of angiosarcoma. In this regard, we showed that 11 out of the 13 cases of human angiosarcoma showed positive result with anti-TCL1b and anti-phospho-Akt. Functionally, ‘TCL1b-*Akt-in'* effectively inhibited the cellular proliferation of sarcoma cells. It is plausible that TCL1b possibly serves as a putative therapeutic target for angiosarcoma, a rare form of human neoplastic disease with poor prognosis. Furthermore, it is possible that suppression of TCL1b isoform could serve as a potential therapeutic target for various human neoplastic diseases. Further studies are required to investigate this possibility.

## Materials and methods

### Co-immunoprecipitation experiments

Co-immunoprecipitation experiments were essentially described previously.^12, 53^ Briefly, 293T cells (ATCC) were transfected with a total of 9 μg of indicated plasmids: pUC-CAGGS-Flag-TCL1b with pCMV6-HA-Akt2. Seventy-two hours after transfection, cells were washed twice with ice-cold phosphate-buffered saline (PBS) and lysed with ice-cold Brij 97 lysis buffer. Lysates were precleaned with protein G/protein A beads mixture (50% v/v, GE Healthcare, Uppsala, Sweden) for 1 h, immunoprecipitated with anti-HA (12CA5, Boehringer Manheim, Indianapolis, IN, USA) or anti-Flag antibody (F3165, Sigma, St Louis, MO, USA) with anti-mouse IgG (X0931, Dako, Glostrup, Denmark) as a control, run on sodium dodecyl sulfate–polyacrylamide gel electrophoresis and immunoblotted with HRP (horse radish peroxidase)-conjugated anti-HA antibody (3F10, Boehringer Manheim) or HRP-conjugated anti-Flag antibody (A8592, Sigma).

### Endogenous interaction of TCL1b with Akt using co-immunoprecipitation assays

COS-7 cells (ATCC) were cultured in the presence of 10% FBS in DMEM. The cells were washed twice with ice-cold PBS, lysed with ice-cold Brij 97 lysis buffer in which Tris-HCl was replaced with PBS (pH 7.4) and proteinase inhibitors (leupeptin and AEBSF), and treated with DSP (2 mM, Pierce, Rockford, IL, USA) for 30 min, precleaned with protein G/protein A mixture (50% v/v, GE Healthcare) for 1 h, immunoprecipitated with anti-Akt (Cell Signaling Technology, Beverly, MA, USA) or control antibody (mouse IgG), added additional DTT 50 mM to cleave DSP, (lane 5), resolved onto sodium dodecyl sulfate–polyacrylamide gel electrophoresis (15% Tris glycine gel) and immunoblotted with the anti-Akt antibody (Cell Signaling Technology, left panel) or anti-TCL1b antibody (right panel). Expression levels of Akt (lane 1) or TCL1b (lane 4) were also shown by using immunoblot as input.

### *In vitro* kinase assays

*In vitro* kinase assays (IVK) were performed as described previously^12^ with minor modifications. TCL1b and GSK-GST fusion proteins were generated according to the manufacturer's protocol (GE Healthcare), except using Brij 97 lysis buffer. Akt from 293T cells transfected with pCMV6-HA-Akt or non-transfected 293T cells as a control was immobilized with the anti-HA antibody. IVK reaction was performed using GSK (CGPKGPGRRGRRRTSSFAEG)-GST fusion protein (2.0 μg) as substrate or GST-fusion proteins as a control by incubating a total of 30 μl in the presence of 5 μl of immobilized Akt in the presence of 9.6 μg of GST (control) or TCL1b recombinant proteins for the indicated 0–3 min (for time-course studies) at 25 °C. For dose-escalation studies, TCL1b proteins (0–6.4 μg) in the presence or absence of 5 μl of immobilized Akt for 7 min at 25 °C. The reaction was terminated by adding sodium dodecyl sulfate sample buffer at indicated time points.

### Cellular Akt phosphorylation

293T cells (ATCC) were transfected with a total of 3 μg per 10-cm dish of the indicated plasmids: sham (vector control), TCL1b, TCL1 or Myr-Akt (HA-Myr-PH (4-129) human-Akt1 in the pECE vector^54^). After the transfection, cells were serum-starved for 16 h and lysed, and an equal amount of protein was loaded into individual lanes and western blot was performed using indicated antibodies.

### Agilent expression array analysis

Human TCL1b, TCL1 in pFlag-CMV2-vector,^12^ Myr-Akt (HA-Myr-DPH (4-129)human-Akt1 in pECE vector^54^), a constitutively active form of Akt, or pBluescript (empty vector) as an internal control (total 3.0 μg of indicated plasmid DNAs per 10-cm dish) were transfected into 293T (ATCC) cells by calcium phosphate transfection. After 24 h of transfection, the cells were serum-starved for 16 h and mRNAs were isolated using RNA isolation kit (NucleoSpin RNA II, MACHEREY-NAGEL no. 740955.10) for microarray-based genomic analysis using Agilent Array Expression Analysis system. Quantification and quality control of RNA were conducted by Agilent 2100 Bioanalyzer (Agilent, Santa Clara, CA, USA). Transcripts of either over two-fold increase (upregulation) or two-fold decrease (downregulation) compared with pBluescript-transfected cells as a baseline control were considered significant and selected for further analysis. Raw data from the DNA microarray are provided in the Supplementary Data (Supplementary Data Table 1).

### Soft agar colony-transformation assay

The colony-transformation assay was performed as described elsewhere. Briefly, 5 μg per 10-cm dish of human TCL1b, TCL1 in pFlag-CMV2-vector,^12^ Myr-Akt (HA-Myr-ΔPH (4–129) human-Akt1 in pECE vector^54^), a constitutively active form of Akt, or pBluescript as control were transfected into NIH3T3 (ATCC) cells using lipofectamine LTX (Invitrogen, Carlsbad, CA, USA). The cells (0.75 × 10^6^) were transferred to six-well plates in soft agar, incubated at 37 °C for 21 days and the numbers of the colonies were counted using microscope at × 40 magnification (Olympus, Tokyo, Japan, BX50).

### Generation of TCL1b-transgenic mice

We generated transgenic mice for the human *TCL1b* gene controlled by the CMV-β-actin promoter on C57BL/6 background. Human full-length TCL1b was subcloned into pUC-CAGGS-TCL1b^31^ vector, digested with SalI, and resulting SalI fragment was isolated for creating transgenic mice. All mice were housed under conventional barrier protection in accordance with the Institute for Genetic of Medicine of Hokkaido University guidance approved by the animal care committee.

### Isoform-specific TCL1 protein polyclonal antibodies

As no anti-TCL1b antibody that specifically recognizes endogenous TCL1b expression is readily available,^55^ we generated polyclonal anti-sera that specifically recognize isoform-specific protein of TCL1 family proteins (Supplementary Data Figure S5). Isoform-specific TCL1 family polyclonal antibodies were generated by using GST-fused full-length human TCL1/TCL1b/MTCP1 (GE Healthcare) as an immunogen.

### Immunohistochemistry for mouse tissues

Paraffin-embedded tissues were sectioned into 3-μm-thick sections. Sections were deparaffinized and treated with Peroxidase-Blocking Solution (Dako) for 5 min. Antigen retrieval was performed in 10 mM citrate buffer (pH 6.0) at 97 °C for 20 min in a water bath. Primary antibodies were diluted 1:500 for TCL1b, 1:25 for phospho Akt antibodies (Cell Signaling Technology no. 4051) and 1:200 for VEGFR2 with Antibody Diluent (Dako). Slides were incubated with primary antibodies for TCL1b and VEGFR2 for 30 min at room temperature or for p-Akt overnight at 4 °C. Slides were incubated with Histofine Simple Stain Mouse MAX-PO(R) (Nichirei, Tokyo, Japan) for TCL1b, EnVision (Dako ChemoMate, Dako) for VEGFR2 antibody, or histofine mouse stain kit (Nichirei, no. 414321) for anti-phospho-Ser473 Akt (Cell Signaling Technology no. 4051) for 30 min at room temperature. Immunoreactivity was visualized with 3,3′-diaminobenzidine.

### Immunostaining of human-derived samples

Human cancer panel (SuperBioChips laboratories) and angiosarcoma samples were immunohistochemically stained using anti-TCL1b or anti-phospho-Ser473 Akt (Cell Signaling Technology, no. 4060) antibodies. Immunohistochemical staining was performed essentially the same way as described above, except by using EnVision (Dako ChemoMate, Dako) as a secondary antibody system for both antibodies.

### Flow cytometry detection for the phosphorylation of Akt in retroviral vector-transduced cells

Retroviral transductions were carried out by using the Phoenix Retroviral Expression System (Orbigen, San Diego, CA, USA). To generate recombinant retroviruses, the pBMN-GFP-TCL1b-Akt-in or pBMN-GFP plasmids were transfected into Phoenix-Eco cells by the calcium phosphate method. The retroviral supernatant was harvested at 48 and 72 h after transfection and was used for infection of the cells. NIH3T3 cells (ATCC) were cultured with retroviruses for 72 h. The cells were collected with ice-cold PBS, 3 mM EDTA, fixed by PBS containing 3.7% formaldehyde and permeabilized by 90% methanol. Then, the cells were rinsed by using FACS buffer (PBS–0.1%BSA–0.1%NaN_3_), blocked by 2.4G2 conditioned media (Fc block) and stained with Anti-phospho-Akt Thr308 rabbit monoclonal antibody (2965, Cell Signaling Technology), Anti-phospho-Akt Ser473 rabbit monoclonal antibody (4060, Cell Signaling Technology), Anti-Akt rabbit polyclonal antibody (9272) or Isotype Control (Rabbit IgG, Jackson, Bar Harber, ME, USA) with Alexa Fluor-647 goat anti-rabbit IgG monoclonal antibody (A-21245, Molecular Probes, Eugene, OR, USA). The stained cells were resuspended in 0.5 ml of FACS buffer and analyzed using flow cytometer (FACS CantoII, BD Biosciences, San Jose, CA, USA).

### Real-time cell proliferation analysis by electric cell-substrate impedance sensing

Growth potentials of J774.1 (mouse reticulum cell sarcoma) and ISO-S1 (mouse angiosarcoma) were assessed by electric cell-substrate impedance sensing (ECIS Zθ Applied Biophysics, Tory, NY, USA). J774.1 and ISO-S1 cell lines were seeded in 8WCP cell-proliferation plates with 50 μM of TAT-FLAG (NH_2_-YGRKKRRQRRR-DYKDDDK-COOH) or TAT-TCL1b*-Akt-in* (NH_2_-YGRKKRRQRRR-RLGVPPGRLWIQRPG-COOH) peptide. Measurements were performed for 18 h at 8 kHz.

### Cell-proliferation analysis (cell count method)

ISO-S1 (1 × 10^4^ cells) and J774.1 cells (5 × 10^4^ cells) were seeded in 24-well plates with 50 μM of TAT-FLAG (NH_2_-YGRKKRRQRRR-DYKDDDK-COOH) or TAT-TCL1b-Akt-in (NH_2_-YGRKKRRQRRR-RLGVPPGRLWIQRPG-COOH) peptide. Cells were counted using the trypan blue dye exclusion method at 24 and 48 h after peptide treatment.

### Antibodies and reagents used in this study

Anti-Ser 473 Akt for immunostaining (D9E, 4060, Cell Signaling Technology), anti-Ser 473 Akt for immunostaining (587F11, 4051, Cell Signaling Technology), anti-VEGFR2 for immunostaining (A-3, Santa Cruz Biotechnology, Santa Cruz, CA, USA), anti-HA (12CA5, Boehringer Manheim), anti-Flag M2 antibody (F3165, Sigma), anti-Mouse IgG (X0931, Dako), anti-HA-HRP High Affinity (3F10, 2013819, Boehringer Manheim), anti-Flag M2-HRP conjugate (A8529, Sigma), anti-Akt antibody (9272, Cell Signaling Technology) and anti-phospho-GSKα/β (9331, Cell Signaling Technology).
