# Supplementary material for: Protooncogene TCL1b functions as an Akt kinase co-activator that exhibits oncogenic potency in vivo
Source: Oncogenesis. 2013 Sep 16;2(9):e70–. doi: 10.1038/oncsis.2013.30 (PMC3816220; doi:10.1038/oncsis.2013.30)
Supplement: Supplementary Figure S1 [file oncsis201330x1.pdf]

Y(TCL1b) = 0.295X(TCL1) + 0.655Z(Myr-Akt) + 0.072

R= 0.865, P< 0.05

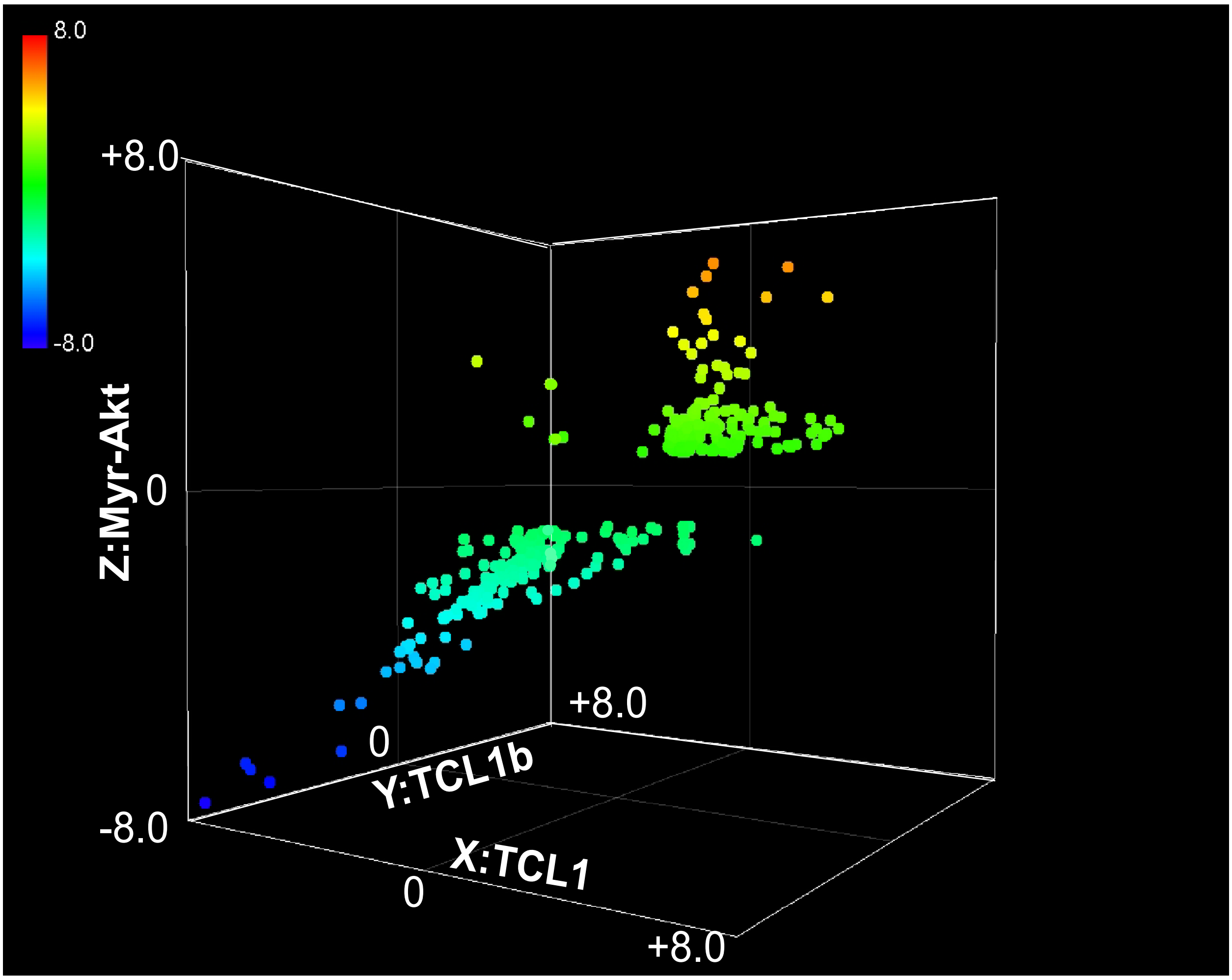

Supplemental data fig-S1.

Multiple regression analysis of the transcripts induce by TCL1b, TCL1, and Myr-Akt

By multiple regression analysis of the transcripts induce by TCL1b, TCL1, and Myr-Akt showed significant correlations with R value of 0.865 ($p<0.05$) for the predicted equation of [Y (TCL1b) = 0.072 + 0.295X (TCL1) + 0.655Z (Myr-Akt)].
