# Supplementary material for: Protooncogene TCL1b functions as an Akt kinase co-activator that exhibits oncogenic potency in vivo
Source: Oncogenesis. 2013 Sep 16;2(9):e70–. doi: 10.1038/oncsis.2013.30 (PMC3816220; doi:10.1038/oncsis.2013.30)
Supplement: Supplementary Figure S2 [file oncsis201330x2.pdf]

A

Myr-Akt

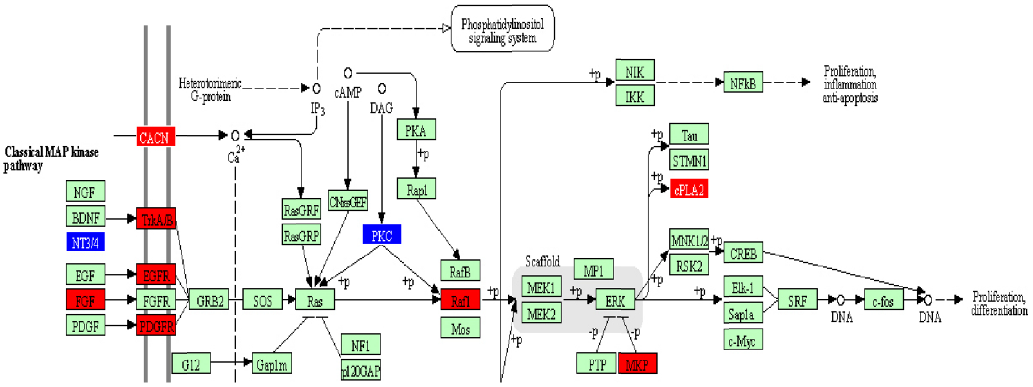

B

TCL1b

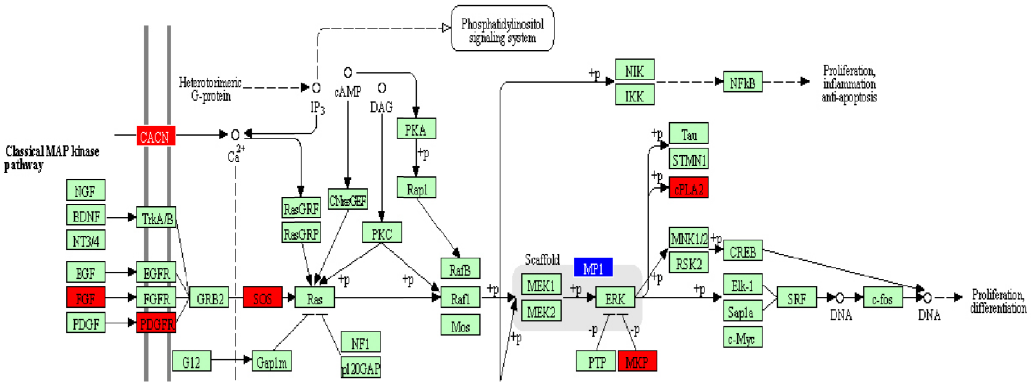

C

TCL1

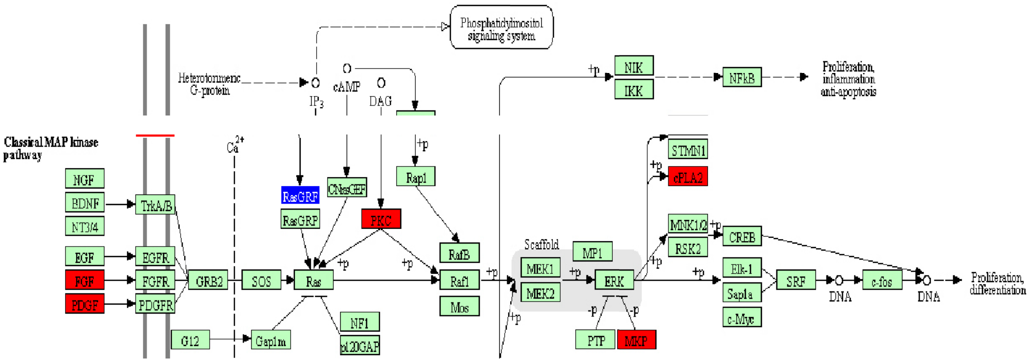

Supplemantal data fig S2A-C.

KEGG pathway mapping represents the process to map molecular datasets, especially large-scale datasets in genomics, transcriptomics, proteomics, and metabolomics for biological interpretation of higher-level systemic functions. We examined the KEGG pathway mapping using mRNA obtained from TCL1, TCL1b, and Myr-Akt transfected 293T cells with pBluescript (empty vector) transfection as a base line control. KEGG pathway mapping analysis showed TCL1b induces similar gene profiles as Myr-Akt or TCL1. In MAPK signal pathway of KEGG pathway mapping that includes 268 transcripts, 24 genes were significantly altered by Myr-Akt (A), 20 genes by TCL1b (B), and 18 genes by TCL1(C).
