# Supplementary material for: Protooncogene TCL1b functions as an Akt kinase co-activator that exhibits oncogenic potency in vivo
Source: Oncogenesis. 2013 Sep 16;2(9):e70–. doi: 10.1038/oncsis.2013.30 (PMC3816220; doi:10.1038/oncsis.2013.30)
Supplement: Supplementary Figure S3 [file oncsis201330x3.pdf]

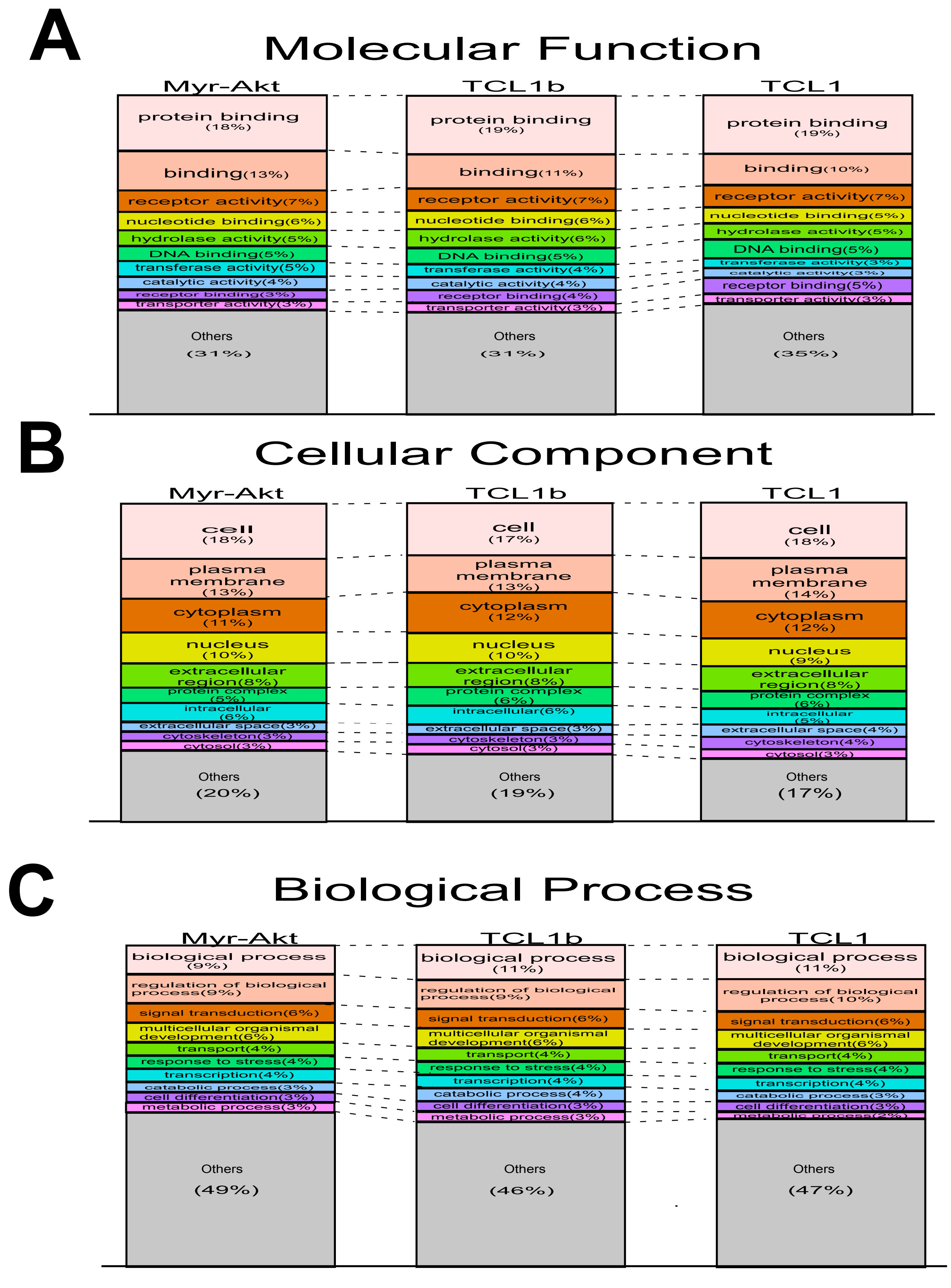

Supplematntal data fig. S3A-C. TCL1b-induced transcripts exhibited similar gene induction profiles in Gene Ontology slim analysis.

Gene Ontology slim (GO slim) provides ontology of defined terms representing gene product properties that are classified into three major categories. Molecular function is defined as the biochemical activity of a gene product (A). Cellular component refers to the place in the cell where gene product is active (B).Biological process refers to a biological objective to which the gene or gene product contributes (C). We performed GO slim analysis using gene transcripts of TCL1b, Myr-Akt or TCL1 with pBluescript transfected cells as a base line control. It is noteworthy that distribution of the profiles of the genes induced by TCL1b- transfected cells represented almost identical to TCL1 or Myr-Akt compared to the transcripts from control cells in all three categories.
