# Supplementary material for: Protooncogene TCL1b functions as an Akt kinase co-activator that exhibits oncogenic potency in vivo
Source: Oncogenesis. 2013 Sep 16;2(9):e70–. doi: 10.1038/oncsis.2013.30 (PMC3816220; doi:10.1038/oncsis.2013.30)
Supplement: Supplementary Figure S4 [file oncsis201330x4.pdf]

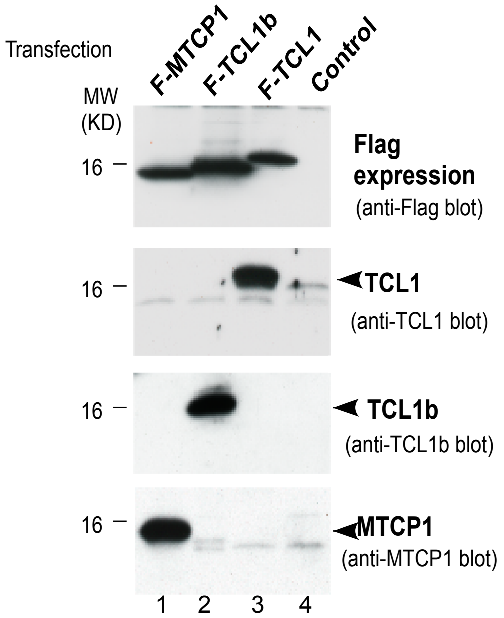

Supplemantal data fig. S4. Polyclonal anti-sera that specifically recognize TCL1 family protooncogene isoforms.

Polyclonal rabbit anti-sera were generated using GST-fused full length human TCL1, TCL1b, or MTCP1 as immunogene. These antibodies specifically recognize each isoform of TCL1 family protooncogene on immunoblot.

Method: 293T cells (ATCC) were transfected with 7.5 µg/ dish of human Flag-tagged TCL1, TCL1b or MTCP1 with pBluescript as a negative control by calcium phosphate transfection. 72 hours later, cells were harvested, washed with ice-cold PBS and lysed with Brij 97 lysis buffer, resolved onto SDS PAGE and immunoblotted by indicated TCL1 family isoform specific rabbit polyclonal antibodies and detected by ECL.
