# Supplementary figures and images for: Protooncogene TCL1b functions as an Akt kinase co-activator that exhibits oncogenic potency in vivo
Source: Oncogenesis. 2013 Sep 16;2(9):e70–. doi: 10.1038/oncsis.2013.30 (PMC3816220; doi:10.1038/oncsis.2013.30)

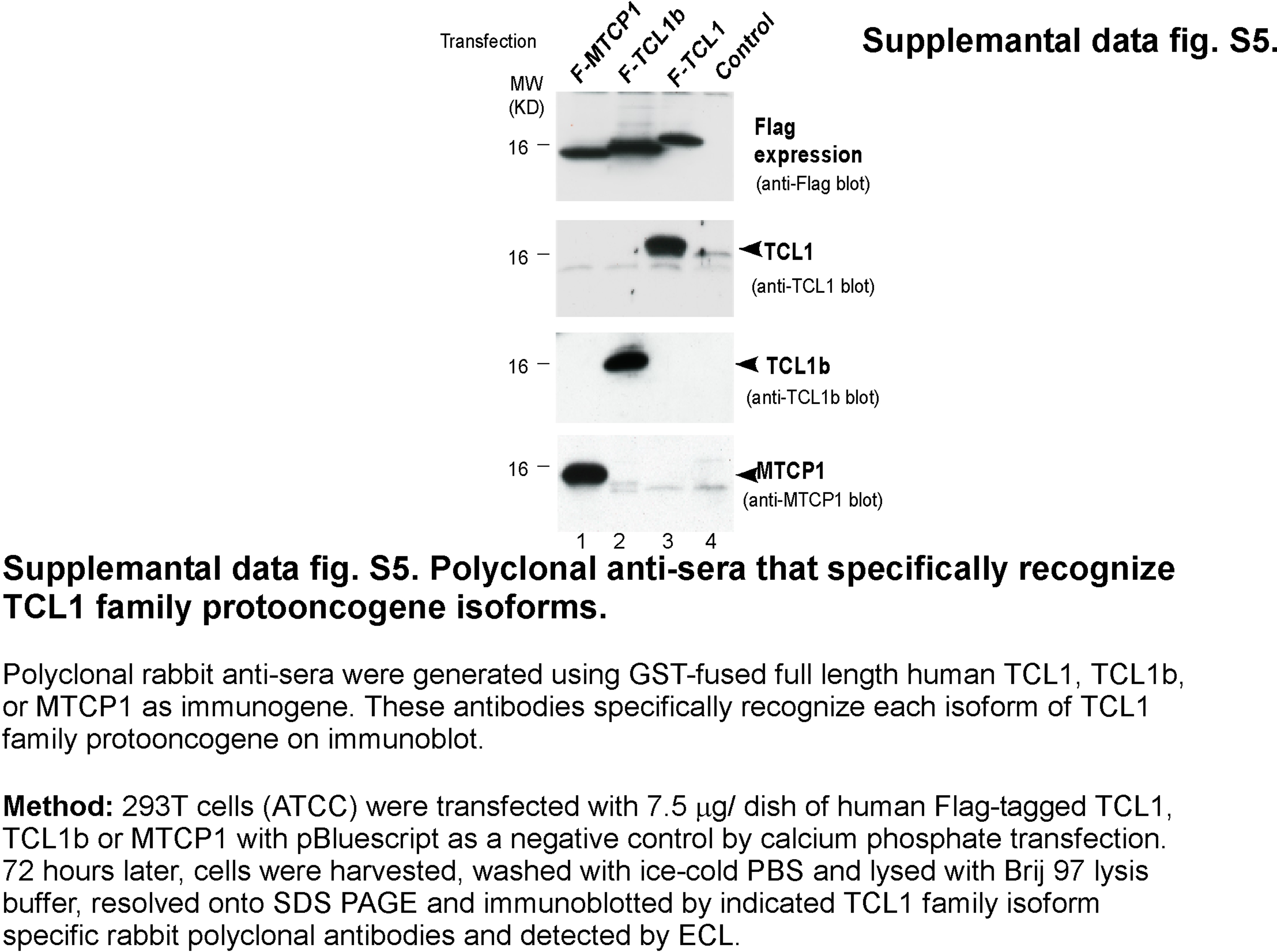

Supplement: Supplementary Figure S5 [file oncsis201330x5.tif]
