# Supplementary material for: Protooncogene TCL1b functions as an Akt kinase co-activator that exhibits oncogenic potency in vivo
Source: Oncogenesis. 2013 Sep 16;2(9):e70–. doi: 10.1038/oncsis.2013.30 (PMC3816220; doi:10.1038/oncsis.2013.30)
Supplement: Supplementary Table and Movie Legends [file oncsis201330x8.doc]

**Legend for Supplemental Table 1.**

Human TCL1b, TCL1 in pFlag-CMV2-vector, Myr-Akt, or pBluescript were transfected into 293T cells. mRNAs were isolated for Agilent Array Expression Analysis system. Quantification and quality control of RNA were conducted. Transcripts of either over two fold increase (up-regulation) or two fold decrease (down-regulation) compared to pBluescript transfected cells as a base line control were considered significant and selected for further analysis. Raw data from the DNA microarray was provided in the supplemental data

.

**Legend for supplemental movie**

By multiple regression analysis of the transcripts induce (or suppressed) by TCL1b, TCL1, or Myr-Akt showed significant correlations with R value of 0.865 (p<0.05) for the predicted equation of [Y (TCL1b) = 0.072 + 0.295X (TCL1) + 0.655Z (Myr-Akt)].
